# Ecological influence of sediment bypass tunnels on macroinvertebrates in dam-fragmented rivers by DNA metabarcoding

**DOI:** 10.1038/s41598-018-28624-2

**Published:** 2018-07-05

**Authors:** Joeselle M. Serrana, Sakiko Yaegashi, Shunsuke Kondoh, Bin Li, Christopher T. Robinson, Kozo Watanabe

**Affiliations:** 1 Department of Civil and Environmental Engineering, Ehime University, Bunkyo-cho 3, Matsuyama, Ehime 790-8577 Japan; 2 Department of Civil and Environmental Engineering, University of Yamanashi, 4-3-11 Takeda, Kofu, Yamanashi 400-851 Japan; 3 Swiss Federal Institute of Aquatic Science and Technology (Eawag), Überlandstrasse 133, 8600 Dübendorf, Switzerland, and Institute of Integrative Biology, ETHZ, 8092 Zürich, Switzerland

## Abstract

Sediment bypass tunnels (SBTs) are guiding structures used to reduce sediment accumulation in reservoirs during high flows by transporting sediments to downstream reaches during operation. Previous studies monitoring the ecological effects of SBT operations on downstream reaches suggest a positive influence of SBTs on riverbed sediment conditions and macroinvertebrate communities based on traditional morphology-based surveys. Morphology-based macroinvertebrate assessments are costly and time-consuming, and the large number of morphologically cryptic, small-sized and undescribed species usually results in coarse taxonomic identification. Here, we used DNA metabarcoding analysis to assess the influence of SBT operations on macroinvertebrates downstream of SBT outlets by estimating species diversity and pairwise community dissimilarity between upstream and downstream locations in dam-fragmented rivers with operational SBTs in comparison to dam-fragmented (i.e., no SBTs) and free-flowing rivers (i.e., no dam). We found that macroinvertebrate community dissimilarity decreases with increasing operation time and frequency of SBTs. These factors of SBT operation influence changes in riverbed features, e.g. sediment relations, that subsequently effect the recovery of downstream macroinvertebrate communities to their respective upstream communities. Macroinvertebrate abundance using morphologically-identified specimens was positively correlated to read abundance using metabarcoding. This supports and reinforces the use of quantitative estimates for diversity analysis with metabarcoding data.

## Introduction

Catchment-based transformations from anthropogenic channel modifications such as reservoir and dam construction alter the physical, chemical and biological structure and function of rivers^1,2^. In particular, downstream changes below reservoirs (dams) in hydrology and sediment regimes directly impact macroinvertebrate assemblages^3,4^. Furthermore, dams are physical barriers that obstruct dispersal and migration of stream macroinvertebrates^5^ and fish^6^. A sedimentation management strategy for various reservoirs in Japan, Switzerland and Taiwan involve the use of sediment bypass tunnels (SBTs). SBTs are used primarily to maintain an active storage capacity of the reservoir, but can also serve to replenish sediment in downstream reaches below dams that are often sediment depleted. Bypass tunnels route sediments around the dam into tail waters, thus reducing sediment accumulation in reservoirs and adding sediment to residual flow reaches below dams^7^. Enhanced sediment supply through SBTs can potentially improve sediment conditions of downstream river channels^8,9^ and facilitate macroinvertebrate diversity through changes in sediment properties^10^. Sediment characteristics of riverbeds such as size, surface roughness, compactness and suitability are important physical determinants of macroinvertebrate diversity and distribution^11–13^.

Some recent studies reported a positive influence of SBT operations on downstream reaches of dam-fragmented rivers using macroinvertebrate indices to assess ecological status. For example, Martín *et al*.^14,15^ showed that SBT operations in a Swiss reservoir acted as short-term disturbances to receiving waters, enhancing sediment connectivity and flow variability, thus promoting biotic changes in macroinvertebrate assemblages over time. Further, Kobayashi *et al*.^8,16^ observed a recovery in macroinvertebrate taxa richness and community composition downstream of Asahi Dam, Japan, after 2–3 years of SBT operation. Likewise, Auel *et al*.^17^ reported that macroinvertebrate richness and composition downstream of dam-fragmented rivers increased with increasing SBT operation time. Estimates of macroinvertebrate diversity in these studies were done using morphological identifications mainly categorized at the functional feeding group^17^, order^14^, or genus level^16^.

Traditional morphology-based macroinvertebrate assessments are constrained not just by time and survey costs, but more importantly by the large number of morphologically cryptic, relatively small-sized and undescribed species^18^, resulting in coarse taxonomic level identifications^19^. To mitigate these limitations, DNA metabarcoding has been used successfully and proven faster, less expensive, and more accurate for evaluating biotic diversities and distributions at higher taxonomic resolution than morphology-based assessments^20–23^. For example, studies have demonstrated the use of metabarcoding as a precise and alternative tool to estimate arthropod diversity and composition^18,24–27^. Metabarcoding may be an efficient and effective means for assessing SBT operations on receiving streams, but has yet to be tested.

In this study, we used DNA metabarcoding to assess macroinvertebrate diversity and composition to better our understanding of the ecological consequences of SBT operations on riverine ecosystems. We hypothesized that SBT operations improve sediment conditions in residual reaches below dams, thereby positively effecting macroinvertebrate assemblages. To test this hypothesis, we sampled three dam-fragmented rivers with SBTs in Switzerland with different operation time spans (2 to 92 years) and operation frequencies (1–10 days/year to ca. 200 days/year). For comparison, we also assessed macroinvertebrate assemblages in dam-fragmented rivers without SBTs and free-flowing rivers as reference sites. We used metabarcoding data from macroinvertebrate larval samples to identify species, estimate richness and diversity, and calculate community dissimilarities between upstream and downstream points in each river.

## Results

### Taxonomic identification

A total of 6,853 macroinvertebrate larvae were collected from 16 sampling sites at 7 rivers. Morphological identification assigned specimens into 24 insect families (morpho-families) within 4 orders. Ephemeroptera comprised 63% of the specimens collected, followed by Diptera (22%), Plecoptera (11%), and Trichoptera (4%). Baetidae was the most dominant morpho-family at 52%, followed by Chironomidae, Simuliidae, and Heptageniidae, representing 11%, 10% and 10% of the collected specimens, respectively. For the metabarcoding analysis, 2,355,940 (44%) sequences from 5,810,864 raw forward reads were retained after quality filtering. From this, 1,908,508 reads were mapped into 1,222 operational taxonomic units (OTUs) excluding chimeric and singleton sequences (Table 1). BLASTn searches matched 322 representative OTUs (50.7% of reads) on BOLD, and 417 (39.6% of reads) on GenBank. The remaining 483 OTUs either have <97% sequence similarity (9.6% of reads) or have no match (0.13% of reads) on the database. In total, 739 OTUs (90.3% of reads) were taxonomically identified at the species level having a > 97% identity threshold. Five of the taxonomically assigned OTUs are non-arthropod sequences (0.01% of reads), e.g. *Leptolegnia* sp., and were discarded in the subsequent analyses. From the remaining 734 OTUs, a total of 8 insect orders, 36 families, 65 genera, and 131 species were represented in the metabarcoding data.

**Table 1:** Summary of metabarcoding data, taxonomic identification and species richness across 16 sampling sites.

Rivers	Sites	Sample Size	Morpho-family^†^	Metabarcoding Reads			Arthropod OTUs	Metabarcoding Tax. ID	
				Raw	Filtered	Mapped to Arthropod OTUs		Families	Species (Richness)
*Dam-fragmented*									
Burvagn Dam (Gelgia River)	US	621	14	277,528	132,964	107,655	458	15	60
	DS	575	9	500,652	237,728	182,147	508	16	65
Isenthal Dam (Isenthalerbach)	US	171	11	260,402	124,617	79,831	322	20	60
	DS	821	12	553,363	136,255	117,853	288	13	50
*Dam-fragmented with SBT*									
Pfaffensprung Dam (Reuss River)	US	641	12	253,254	148,241	129,568	293	17	46
	DS-A	1522	10	362,477	161,635	135,275	270	15	41
Egschi Dam (Rabiusa River)	US	288	12	252,696	108,979	87,625	390	15	50
	DS-B	158	12	441,620	159,435	135,880	318	10	36
	DS-A	237	7	315,665	119,695	99,479	228	13	34
Solis Dam (Albula River)	US	575	10	536,536	130,451	107,970	422	15	46
	DS-B	461	9	297,590	170,244	130,527	373	13	43
	DS-A	19	7	352,962	111,087	90,294	229	11	32
*Free-flowing*									
*Nolla River*	US	194	7	424,824	116,326	100,728	279	15	34
	DS	54	7	253,407	137,800	103,591	244	14	38
*Kärstelenbach*	US	301	12	304,706	127,751	108,236	372	17	61
	DS	215	10	423,182	232,732	191,720	354	12	50
**Total**	**n/a**	**6,853**	**24**	**5,810,864**	**2,355,940**	**1,908,379**	**734**	**36**	131

^†^Morphologically identified samples at the family level.

Ephemeroptera was the most prevalent order with 86.7% of reads, followed by Diptera, Plecoptera and Trichoptera with 8.6%, 3.8% and 0.6% of reads, respectively. The orders Coleoptera, Lepidoptera, Odonata and Hymenoptera comprised the remaining 0.25%. Species with the most abundant sequences were *Baetis alpinus* (46%) and *Baetis rhodani* (13%). Species with >2% sequence abundance were *Ecdyonurus venosus* (8%), *Rhithrogena* sp. 28 (5%), *Rhithrogena* sp. 14 (4%), *Rhithrogena* sp. 19 (4%), *Simulium argyreatum* (3%), *Simulium variegatum* (3%) and *Ecdyonurus submontanus* (2%). Mayflies and simuliid blackflies were the most represented taxa in the metabarcoding data, and were present at all sites regardless of river site type. These species showed relatively dissimilar abundances between the upstream and downstream sites of dam-fragmented rivers, and relatively similar abundances at free-flowing sites. Accordingly, Pfaffensprung followed the pattern observed for free-flowing sites, whereas Egschi and Solis followed the pattern observed at dam-fragmented sites (except for *Baetis alpinus* in Egschi). See Supporting Information (Fig. S1) for the proportional taxonomic abundance of the metabarcoding-identified taxa at the family and species level for each sampling site. However, paired sample t-test revealed no significant difference between the relative abundance of the 9 species with >2% sequence abundance between the US and DS (DS-A/DS-B) sites when grouped based on river categories, except for *Baetis rhodani* with significant difference between the upstream (US) and downstream sites before the SBT outlet (DS-B) (*p* = 0.004) (Fig. S2).

### Diversity and community composition

**Figure 1:**
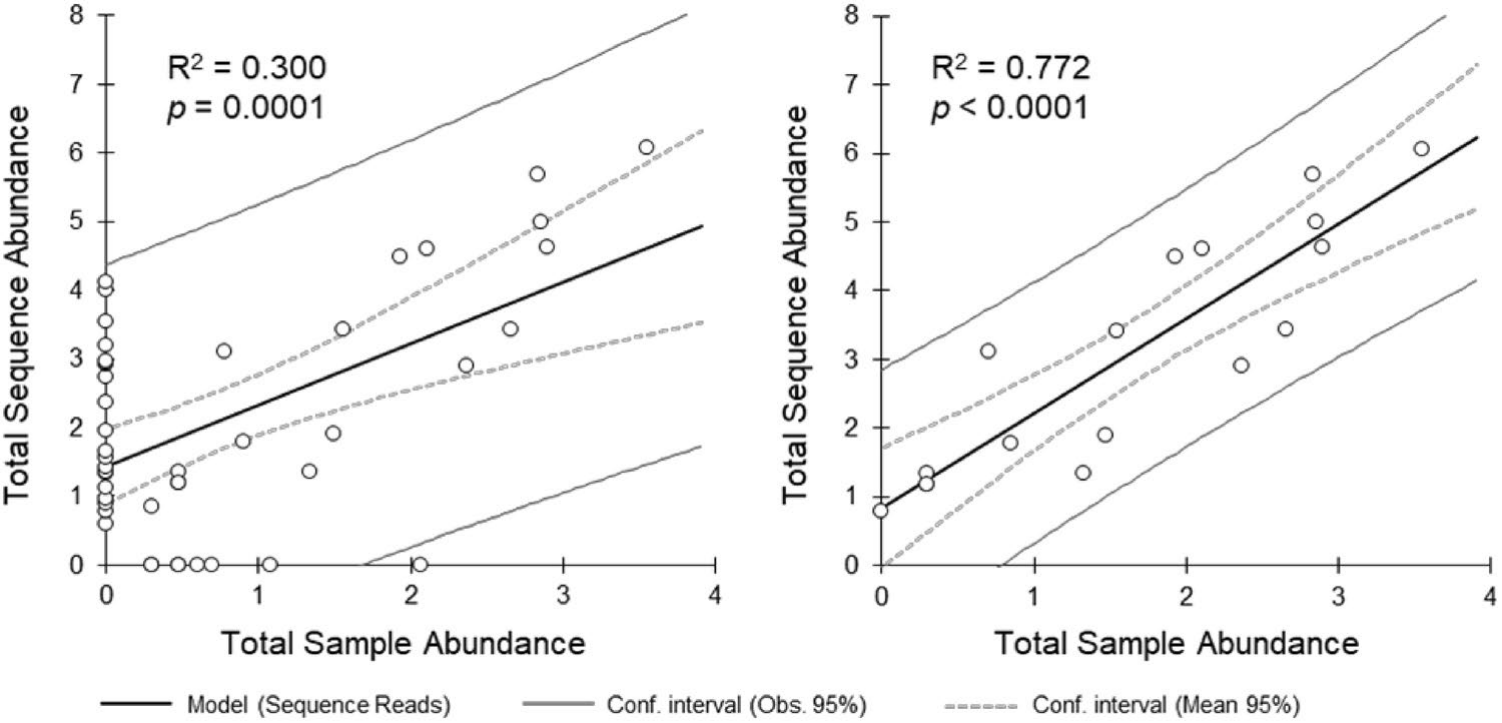
Relative logarithmic sample abundance (morphologically identified families/morpho-families) plotted against the relative logarithmic sequence/read abundance (metabarcoding-identified taxa at the family level) of all the sites via linear regression analysis. Showing analysis including (left) and excluding (right) false positive and false negative detection. Please see Fig. S2 for the correlation results per site.

A positive correlation was found between the total abundance of morpho-families and read abundance of metabarcoding-identified taxa (family level) for all sites, both in analysis including (R^*2*^ = 0.30; *p* = 0.0001) and excluding (R^*2*^ = 0.77; *p* < 0.0001) false positive and false negative detections (Fig. 1). Positive linear correlations (*p*-value < 0.05) were also found for each sampling site including false positive and false negative detections, except for Egschi downstream before the SBT outlet (DS-B) (R^*2*^ = 0.20; *p* = 0.108), Egschi downstream after the SBT outlet (DS-A) (R^*2*^ = 0.28; *p* = 0.052), and Solis upstream (US) (R^*2*^ = 0.15; *p* = 0.090) (Supporting Information Table S1 and Fig. S3). Metabarcoding detected 16 of the 24 morpho-families, representing 98.3% of the reads that accounted for 98.0% of the morphologically identified specimens. The remaining 8 morpho-families (2.0%) not detected by metabarcoding had fewer than 11 specimens, except for Ameletidae (115 individuals). Moreover, metabarcoding detected 20 false-positive families, representing 1.7% of the reads.

Alpha diversity was evaluated using the Simpson’s diversity index (1-D) that reflects the richness, and equitability or evenness of population abundances within a sample of the communities. With values between 0 to 1, the higher the score, the more diverse the community is considered to be. Simpson’s diversity index calculated for each site ranged from 0.80–0.94 (Fig. S4). Dam-fragmented river sites had relatively higher Simpson’s diversity index values (0.93–0.94) compared to free-flowing river sites (0.86–0.89). For dam-fragmented sites with SBTs, upstream and downstream sites at Pfaffensprung had the lowest D (0.80–0.82) of all study sites, while Egschi had 0.83–0.87. Estimated Simpson’s diversity index for Solis dam (0.89–0.92) was relatively higher compared to other dam sites with SBT and free-flowing sites. However, one-way analysis of variance (ANOVA) showed no significant difference [F (6, 15) = 1.15, *p* = 0.28] between the estimated Simpson’s diversity of each sites when grouped based on site type, i.e. dam-fragmented (US and DS), dam-fragmented with SBTs (US, DS-B and DS-A) and the free-flowing (US and DS) sites.

**Figure 2:**
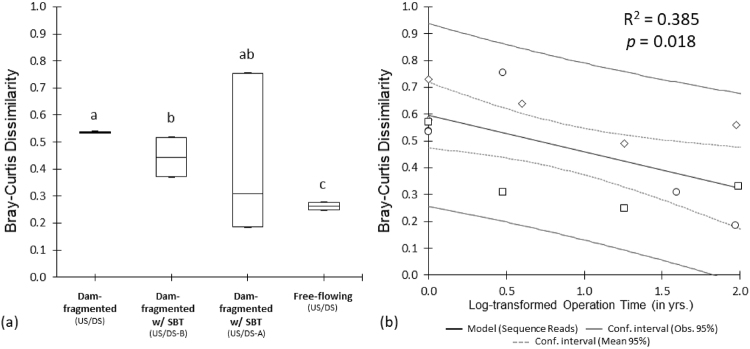
(**a**) Beta diversity (Bray-Curtis dissimilarity) of macroinvertebrate communities between the up- (US) and downstream (DS) sites of dam-fragmented and free-flowing rivers, and between the up- (US) and downstream after the SBT outlet (DS-B/DS-A) sites of dam-fragmented rivers with SBT. Bars without shared letters indicate significant difference (*p* < 0.05). (**b**) Pearson correlation between the community dissimilarity of the US and DS/DS-A sites of dam-fragmented rivers with and without SBTs against log-transformed operation in years (○: n = 5). Invertebrate Bray-Curtis dissimilarity adopted from Kobayashi *et al*.^8^ (□: n = 4) and Auel *et al*.^17^ (◇: n = 5) were included in the analysis.

Bray-Curtis dissimilarity was calculated to measure the dissimilarity between the species composition or beta diversity of the upstream and downstream sites within each study river based on relative sequence abundance. Dissimilarity values ranges from 0 to 1, with the latter score being highly dissimilar. Estimated Bray-Curtis dissimilarity values (Fig. S4) of the dam-fragmented rivers, i.e. Burvagn and Isenthal, were 0.54 and 0.53, while free-flowing rivers, i.e. Nolla and Kärstelenbach, had 0.25 and 0.28, respectively. For dam-fragmented rivers with SBTs, Pfaffensprung had the lowest dissimilarity value (0.18) compared to all study rivers. Dissimilarity for Egschi dam was substantially low (0.37 for US/DS-B; and 0.31 for US/DS-A) in comparison to dam-fragmented rivers without SBTs. The 0.52 dissimilarity value observed between Solis’ US and DS-B sites was considerably similar to values observed for dam-fragmented rivers without SBTs, while its US and DS-A sites had the highest dissimilarity value (0.73) of all rivers. ANOVA was performed to assess the difference between Bray-Curtis values of dam-fragmented, dam-fragmented with SBTs (US/DS-B and US/DS-A) and the free-flowing river sites. The analysis showed a significant difference [F (3, 86) = 11.29, *p* < 0.0001] in the mean values of the jackknife replicates of the Bray-Curtis dissimilarities. Dam-fragmented, free-flowing and dam-fragmented with SBT (US/DS-B) rivers were significantly different, while the dissimilarity between the up- and downstream communities after the outlet of dam-fragmented rivers with SBT (US/DS-A) was not significantly different from the other dam-fragmented sites (Fig. 2a). A significant Pearson correlation between the Bray-Curtis dissimilarity values and SBT operation was observed (R^2^ = 0.385; *p* = 0.018). The dissimilarity between the macroinvertebrate communities of the up- and downstream sites decreased with increasing SBT operation (Fig. 2b).

### Sediment analysis

Here, we present the *D*_*90*_ and *D*_*m*_ as representative grain size parameters for a gravel bed. See Table S2 and Fig. S5 for detected sediments and computed sediment characteristic grain size distributions for each site. For dam-fragmented rivers without SBTs, sediment size in Burvagn for US (*D*_*90*_ = 178 mm; *D*_*m*_ = 78 mm) and DS (*D*_*90*_ = 195 mm; *D*_*m*_ = 82 mm) sites were not significantly different, while Isenthal had significantly finer sediments DS (*D*_*90*_ = 185 mm; *D*_*m*_ = 65 mm) than US (*D*_*90*_ = 187 mm; *D*_*m*_ = 91 mm). Observed *D*_*m*_ among free-flowing rivers was not significantly different (range = 47–90 mm), while *D*_*90*_ values showed finer sediments for US (range = 28–111 mm) than DS (range = 121–205 mm) sites. For dam-fragmented rivers with SBTs, downstream (DS-B and DS-A; range = 69–219 mm) sediment size was relatively finer or similar to their respective US sites (range = 105–249 mm).

## Discussion

We used DNA metabarcoding analysis to assess the influence of sediment bypass tunnel (SBT) operations on the diversity and community composition of macroinvertebrates in dam-fragmented rivers along with a concurrent assessment of reference river sites for baseline comparison. We observed significantly high dissimilarity values between upstream and downstream communities of dam-fragmented rivers compared to free-flowing rivers without dams. Our results support the observed negative effect of reservoir and dam construction on macroinvertebrate diversity and community composition from previous studies^28,29^.

Macroinvertebrate communities on the Reuss (Pfaffensprung dam) and Rabiusa (Egschi dam) rivers with SBT dams showed considerably low dissimilarity compared to dam-fragmented rivers, and were relatively similar to free-flowing rivers. This supports the positive influence of SBT operations on macroinvertebrate assemblages below dams. Alongside dam completion, SBT operation at Pfaffensprung started in 1922 with a discharge capacity of 220 m^3^/s. The SBT is operated to transport as much as several thousand m^3^ of sediment for ca. 200 days/year^30^. Ninety-two years of operation and a high operation frequency per year could be a major reason for the improvement of downstream communities close to the state upstream. Likewise, Egschi’s SBT has been operational for 38 years (1976–2014) with an approximate sediment discharge of 15% from the reservoir by 1984, and a continuous sediment discharge of 50 m^3^/s for 10 days/year^30^. Dissimilarity observed between the upstream and downstream sites above the SBT outlet was higher in comparison to values observed between the upstream and downstream sites below the SBT. This finding could be due to better environmental conditions below compared to above the SBT outlet. Related studies also reported a positive influence of SBT operations on macroinvertebrate e community composition in downstream reaches^14,16,17^. Specifically, Auel *et al*.^17^ observed macroinvertebrate community composition using the Bray-Curtis similarity index (based on samples categorized at feeding groups) in upstream and downstream reaches at a dam with SBT to increase in similarity over time since operation.

On the other hand, observed community composition of macroinvertebrates in the Albula (Solis dam) were relatively similar to dam-fragmented sites (between US and DS-B points), or even greater (between US and DS-A points). This suggests that SBT operation in Solis resulted in little biotic improvement of communities downstream before the SBT outlet, and may even have a negative effect on communities downstream of the SBT. Solis SBT has been operational for 2 years with a discharge capacity of 170 m^3^/s operating 1–10 days/year^30^. Accordingly, previous studies conducted in Solis also reported lower Bray-Curtis similarity between the upstream and downstream communities in comparison to Asahi dam with 17 years SBT operation, but higher than Koshibu dam with a SBT that has not been operational upon survey^16,17^. Likewise, Kobayashi *et al*.^16^ found no evidence of improvement in invertebrate assemblages at Miwa dam, Japan, with an SBT operating for almost 7 years (at the time of study) with a discharge capacity of 300 m^3^/s operating 1–2 times a year. Large volumes of sediment have been mobilized in the downstream reach of Solis dam resulting in pronounced morphological changes in some areas^9^. However, water and sediment scouring or deposition from SBT operation disturbed sediment respiration, periphyton biomass and macroinvertebrate richness downstream^14,15^. Detectable alterations in macroinvertebrate communities are expected following disturbances in downstream reaches, but moderate sediment deposition, good water quality^31^, and stable flows^32^ would more likely influence its recovery. However, due to the minimal operation time of SBTs per year, it requires several years to cause a rejuvenation of geomorphic and hydrologic processes^9^, such as those observed following experimental floods^33^ that promoted the recovery of downstream macroinvertebrate communities near to respective upstream communities.

In addition, SBT operation conditions, frequency and duration influence changes in riverbed conditions; i.e. channel incision and riverbed armoring that subsequently restore natural geomorphic processes in the downstream reach^9^. Riverbed conditions and macroinvertebrate communities in residual channels improved in the years after SBT operation due to increased bed mobility (high flows from SBTs), which is important for the reestablishment of more natural invertebrate communities^8^. In addition, maximum SBT discharge, sediment released through the tunnel, and distance to the outlet mostly influenced hyphoreic properties; i.e. sediment respiration, organic matter content, and sediment size^14^. These factors may also influence assemblages and possible recovery of macroinvertebrate taxa downstream. Flood events and sediment loading to downstream reaches contain substantial particulate organic matter. In particular, food available for filter-feeding invertebrates could increase above intrinsic levels and promote expansion of populations^34^. A decrease in habitat availability, i.e. coarse substrate and associated interstices, directly affects benthic macroinvertebrates from the aquatic insect orders Ephemeroptera, Plecoptera and Trichoptera (EPT), unlike other generalist taxa with high dispersal ability and generalist feeding habits (e.g. chironomids, burrowing mayflies)^35^. Hence, EPTs are expected to recover and replenish local benthic assemblages in the long-term with the addition of coarser sediments in downstream reaches. Response of the fish community to SBT operations also may have an effect on macroinvertebrates. Since macroinvertebrates serve as a primary food resource for many fish species, a decline or increase in fish numbers directly influence the predation risk to prey, most of which are large macroinvertebrates^34^.

We measured grain size parameters for each riverbed via gravelometric image analysis to assess grain size distribution of US and DS (DS-B/DS-A) sites of the study rivers. SBTs route sediments downstream to reduce sediment accumulation in the reservoir, reducing sediment depletion and degradation downstream^7^. We expected to see relatively similar grain size distribution between US and DS-A sites of rivers with SBTs since coarse sediments are transported through the tunnel^17^. Sediment size distribution at all sites in Egschi were similar. However, both *D*_*m*_ and *D*_*90*_ values of US and DS-A sites of rivers fragmented by Pfaffensprung and Solis dams were dissimilar with coarser sediments upstream. On the other hand, Auel *et al*.^17^ observed that grain size on Pfaffensprung and Solis were coarser downstream after the SBT outlet compared to upstream. Our observed values were relatively different to the reported target grain size of each SBT (See Table S3 for the sediment bypass tunnel specifications), and dissimilar with the grain size distribution of other studies. The surface material images we analyzed in this study were taken on the river bank, which might lead to a misrepresentation of the riverbed and hence the unexpected grain size dissimilarity of transported sediments.

Our interpretation of the influence of SBT operation based on metabarcoding data was consistent with the results of previous studies that employed traditional morphology-based assessment with samples mainly categorized at the functional feeding group^17^, order^14^, or genus level^16^. We were able to recover arthropod sequences from collected larvae samples representing 131 species, obtain community profiles from 150-bp COI fragments to estimate species diversity, and calculate pairwise community dissimilarity at the species level that would be difficult to detect using traditional morphological methods^36^. A handful of studies have evaluated the reliability of metabarcoding for the assessment of taxonomic composition and diversity of freshwater macroinvertebrates^18,24,37–39^. Metabarcoding data sets are taxonomically more comprehensive and less dependent on taxonomic expertise^37^, thus providing a quicker and more reliable means of identifying organisms at various taxonomic levels, thereby expanding the taxonomic coverage of ecological studies^40^. The efficiency of metabarcoding-based taxonomic assignment strongly relies on the representation of species in the sequence database. We were able to match 50.7% of our metabarcoding reads on BOLD, and 39.6% to GenBank with a total of 90.3% arthropod sequence identification at a > 97% similarity threshold. The high taxonomic match indicates that most of the aquatic insects in Switzerland are registered and well represented in the reference databases, and that metabarcoding-based assessments on these river ecosystems are reliable for further interpretation.

Quantitative estimation using relative abundances is critical for community characterization and assessment of biological indices^41^. Then again, estimating relative abundance based on read counts remains elusive for most PCR-based metabarcoding assessment of biodiversity^42,43^. Previous metabarcoding studies reviewed by Barnes and Turner^44^ reported a positive correlation between species abundance and relative or absolute sequence read abundance. We assessed the reliability of using sequence abundance by testing the relationship between the abundance of morpho-families and the read abundance of the metabarcoding-identified taxa (family level) via linear regression analysis. A significant positive linear correlation was observed between sample and sequence abundance, which validates and reinforces the use of relative sequence abundance for the downstream diversity analysis and interpretation of our metabarcoding data. Our metabarcoding sequences accounted for most of the morphologically-identified specimens (98%). However, there were still undetected morpho-taxa (false negative) that accounted for the remaining 2%. False absence or detection is unavoidable from metabarcoding data^45,46^. Although new Illumina protocols produce improved results, some common issues such as PCR bias and false-positive detection can still arise^25^. Taxa with low sample abundance would yield insufficient amount of template DNA for PCR and consequently fail to be detected in metabarcoding analysis^43^, which may lead to false negative results. In addition, some of these undetected taxa might have low PCR primer compatibility. The low affinity to primers can lead to less PCR amplification^47,48^. On the other hand, 1.7% of the metabarcoding reads (false positive) represented 20 families that were not included in the morpho-taxa identified. False positives might arise from contamination or errors during PCR and sequencing that were not detected from bioinformatics analysis^46,49^. Setting stringent parameters for read quality filtering reduces the possibility of detecting false positives^50^. The 20 families detected by metabarcoding could be due to misidentifications of samples, a result of environmental DNA or from specimen gut contents. Nonetheless, our study had a considerably acceptable rate of detection similar to previous metabarcoding studies that directly compared morphological and metabarcoding data such as Hajibabaei *et al*.^24^ with 74% (17/23 insect species), Elbrecht and Leese^42^ with 83% (43/52 insect taxa), and Brandon-Mong *et al*.^47^ with 80–90% (insect taxa) detection rates.

## Conclusion

We were able to use metabarcoding data from collected macroinvertebrate larvae to identify samples at the species level, estimate richness and diversity, and calculate community dissimilarities between upstream and downstream sites of 7 Alpine rivers. We report a positive influence of SBT operation on macroinvertebrate assemblages downstream of SBT outlets at two rivers (Pfaffensprung and Egschi). On the other hand, dissimilarity values observed for upstream and downstream sites at Solis dam were relatively similar to dam-fragmented sites, or even higher than the other sites. This suggests that SBT operations had little positive influence on macroinvertebrate communities downstream before the SBT outlet, and could possibly have negative effects on communities downstream of the SBT. The SBT in Solis has only been operational for 2 years at the time the study was conducted. SBT operation still acted as a disturbance, and riverbed conditions are yet to recover to support improvement of the macroinvertebrate communities. Hence, our results revealed that ecological effects of SBT operations on macroinvertebrate communities downstream of dam-fragmented rivers were related to the operation time and frequency of SBT events.

In this study, we primarily aimed to promote metabarcoding as a tool for identifying species and profiling biodiversity of freshwater habitats. Further studies are needed to understand how the ecological impacts of SBT operation on macroinvertebrate communities can be translated to develop holistic management strategies for reservoir and SBT operations in the future. We recommend multi-time sampling of the sites to recover seasonal variation in macroinvertebrate composition in future assessments.

## Methods

### Sample collection

**Figure 3:**
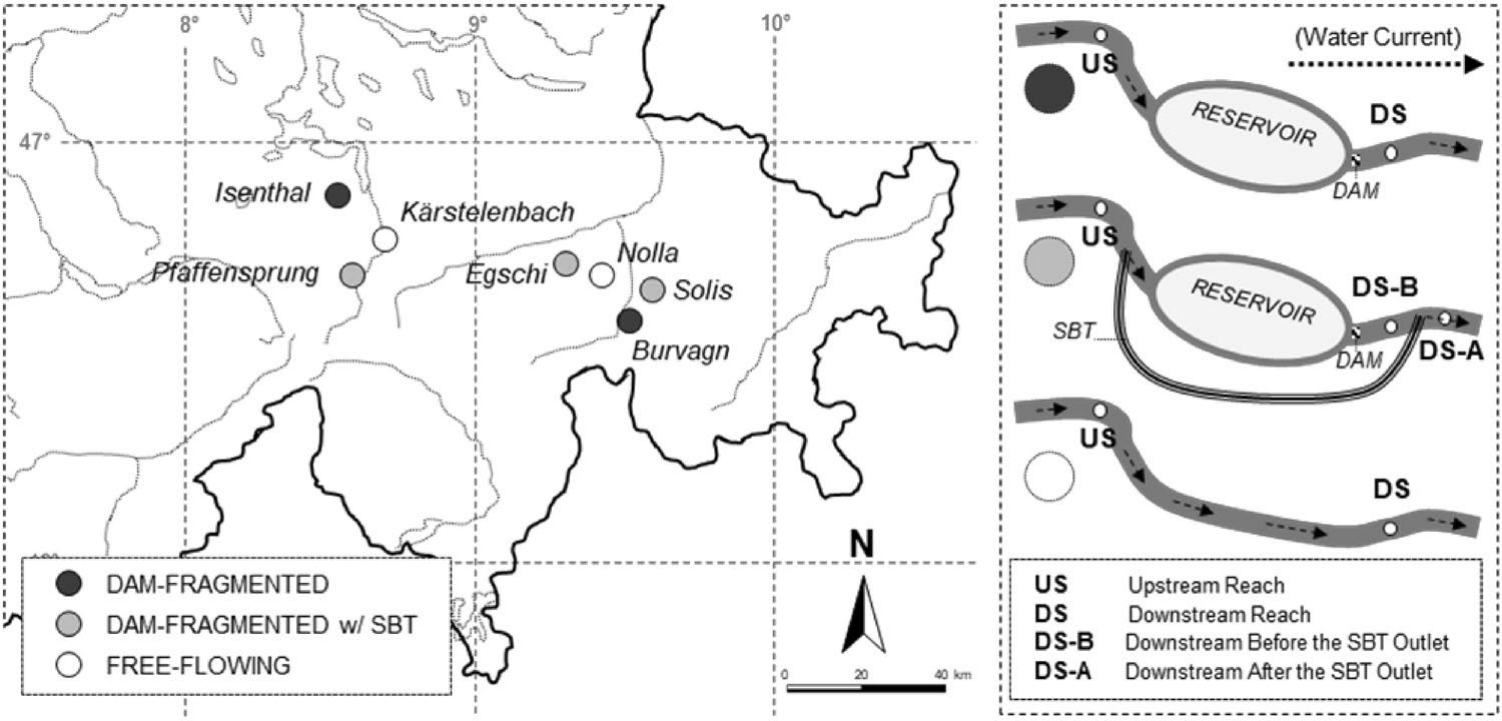
Map of Switzerland showing the location of the two dam-fragmented rivers, three dam-fragmented rivers with SBTs and two free-flowing rivers assessed in this study, and the sites sampled on each study rivers.

The study was conducted in Switzerland in August 2014. Three dams with SBTs were selected for study: Pfaffensprung (46° 42′ 53.22″ N, 8° 36′ 38.46″ E), Egschi (46° 43′ 59.23″ N, 9° 20′ 26.13″ E), and Solis (46° 40′ 41.00″ N, 9° 32′ 4.00″ E) located on the Reuss (Wassen, Uri, CH), Rabiusa (Safien, Grisons, CH), and Albula (Alvaschein, Grisons, CH) rivers, respectively. These rivers have been dam-fragmented for 92, 65, and 28 years, respectively^30^. Three sites were sampled on each river: upstream of the reservoir (US), downstream below the dam but upstream of the SBT outlet (DS-B), and downstream of the SBT outlet (DS-A). Pfaffensprung (Reuss River) was not sampled at point US-B since the SBT outlet was in close proximity to the dam. In addition, two dam-fragmented rivers without SBTs and two free-flowing (non-fragmented by dam) rivers were sampled as positive and negative control rivers, respectively. Dam-fragmented rivers were the Gelgia River (Salouf, Grisons, CH) impounded by Burvagn dam for 65 years (46° 37′ 22.37″ N, 9° 35′ 04.49″ E) and Isenthalerbach River (Flüelen, Uri, CH) impounded by Isenthal dam for 59 years (46° 54′ 36.40″ N, 8° 33′ 50.07″ E). The unregulated Nolla (Thusis, CH) and Kärstelenbach (Silenen, CH) rivers were chosen as free-flowing river sites. All rivers were sampled at upstream (US) and downstream (DS) points of the same stream order at elevations of ca. 800 m a.s.l. (601–911 m a.s.l.), except for the Albula and Gelgia rivers at 1000–1200 m a.s.l. In total, 16 sites were sampled for assessment (Fig. 3). Semi-quantitative samples of macroinvertebrates were collected at each site using a D-frame net (mesh size: 0.5 mm) via the foot-kick method. Samples were preserved in 99.5% ethanol in the field, and replaced twice with fresh 99.5% ethanol upon return to the laboratory. Each sample was sorted and specimens morphologically identified to family using the identification key for the aquatic insects of North Europe by Nilsson^51^ using a stereomicroscope (×112.5).

### DNA extraction, amplicon library preparation and sequencing

Identified macroinvertebrates (family level) at each site were dried, grounded and homogenized. Genomic DNA was extracted using the DNeasy Blood & Tissue Kit (Qiagen, Inc.) following manufacturer instructions. Template DNA were prepared by mixing equal volume of extracted DNA from each tube and each site. The 658-bp fragment of the cytochrome oxidase I (COI) region of the mitochondrial DNA was amplified using the universal Folmer primers - LCO1490 and HCO2198 phosphorylated in the 5′-end^52^. PCR amplification was carried out with a T100TM Thermal Cycler (BioRad Ltd.) in a 40 μl reaction consisting of 3 μl diluted DNA template (10×), 20 μl Phusion® High-Fidelity PCR Master Mix with HF Buffer (New England Biolabs), 2 μl each of the forward and reverse primers (10 μM), and 13 μl of PCR-grade water. PCR cycling conditions were 30 s of initial denaturation at 98 °C, followed by 20 cycles of 10 s denaturation at 98 °C, 30 s annealing at 43 °C, 30 s extension at 72 °C, and a final extension step of 5 min at 72 °C. PCR products were purified by QIAquick PCR Purification Kit (Qiagen, Inc.), and DNA concentration was measured by Quantus™ Fluorometer using the QuantiFluor dsDNA System (Promega). For the amplicon library preparation, 16 index adapter pairs were prepared manually using D700 and D500 Illumina TruSeq adapter sequences. PCR products were adenylated at the 3′-end using *Taq* polymerase. The reaction consists of 0.15–1.5 pmol PCR products, 5 µl of Ex *Taq* buffer (TaKaRa), 3 µl of 25 mM MgCl2 (TaKaRa), 1 µl of 10 mM dATP (Promega), 2.5U Ex *Taq* (TaKaRa), and PCR grade water up to 50 µl. Adenylation was performed in a T100TM Thermal Cycler (BioRad) at 72 °C for one hour. The PCR products were then purified via QIAquick PCR Purification Kit (QIAGEN) and DNA concentration was measured by Quantus™ Fluorometer using the QuantiFluor dsDNA System (Promega). The adapters were ligated to the adenilated products in a 50 µl ligation mixture consisting of 5 µl ligation buffer (TaKaRa), 1 µl T4 DNA ligase (TaKaRa), 40 µl of adenylated product, annealed adapter (100× of mol amount of the adenylated product) and PCR-grade water. Ligation was performed for 19 hours at 16 °C and 65 °C for 2 min in a T100TM Thermal Cycler (BioRad). Ligated products were purified using Agencourt AmpureXP (Beckman Coulter). The amplicon libraries were enriched on a 2nd PCR with a reaction mixture consisting of 5 µl Phusion® High-Fidelity PCR Master Mix with HF Buffer (New England Biolabs), 0.5 µl each of a 10 µM KAPA Illumina primer P1 (5′-AAT GAT ACG GCG ACC ACC GA-3′) and 10 µM Illumina primer P2 (5′-CAA GCA GAA GAC GGC ATA CGA-3′), 3 µl ligated DNA, and PCR-grade water. PCR cycles were 30 s at 94 °C, 15 cycles of 10 s at 94 °C, 30 s at 60 °C, 1 min at 72 °C, a final extension step of 5 min at 72 °C and stored at 4 °C. The PCR products were purified using Agencourt AmpureXP (Beckman Coulter). Amplicon library concentration was quantified by qPCR using the KAPA library quantification kit (KAPA) in a 10 µl reaction volume with 10000× dilutions following the kit’s protocol. The 16 amplicon libraries were normalized to 2 nM and pooled. Finally, 600 μl of a 6 pM denatured pooled library with PhiX (Illumina, final concentration 30%) was prepared, and a 300-bp paired-end sequencing was performed using the MiSeq Reagent Kit v3 (Illumina, Inc.) as per manufacturer instructions.

### Data processing

Paired-end sequencing generated a total of 11,621,728 raw reads. Reverse reads were discarded due to low quality. The remaining 5,810,864 forward reads with an average length of 235-bp, ranging from 35 to 301-bp in length, were treated as unpaired for subsequent analysis. Read quality was checked using FastQC v0.11.5^53^. Non-biological sequences, i.e. primer and index sequences, were trimmed via Trimmomatic v0.36^54^. The UPARSE pipeline^50^ was followed for quality filtering, implementing the maximum expected error method (maxee) that retains ~50% reads for all sampling points. Reads > 2.0 expected error and <150-bp in length were discarded and the surviving reads were truncated at 150-bp length to obtain globally aligned reads. Reads from the 16 sampling points were then pooled and collapsed into unique sequences using a command in USEARCH v9.2.64^55^. The resulting unique reads were clustered into operational taxonomic units (OTUs) with a similarity cut-off value of 97%, subsequently discarding chimeric and singleton sequences. Taxonomic identification was generated by performing BLASTn searches of each OTU representative sequence on a reference database. BLAST hits >97% in the Barcode of Life Database (BOLD)^56^ was used to classify each sequence. Sequences without matches at 97% similarity with BOLD were then queried to GenBank^57^. Representative OTU sequences without matches, queries with taxonomic assignment <97% identity, and non-arthropod matches were excluded from subsequent analyses.

### Diversity and community composition

The OTU table output from the UPARSE pipeline was used as input data to calculate diversity indices in Quantitative Insights into Microbial Ecology (QIIME)^58^ following the eukaryotic diversity analysis protocol developed by Leray and Knowlton^40^. Prior to estimation of diversity, the OTU table was rarefied to accommodate differences in sequencing depth among sampling sites. A rarefaction analysis was required since non-parametric and parametric estimates are sensitive to sample size^40,59^. A paired sample t-test was performed to compare the relative abundance of species with >2% sequence abundance between the US and DS (DS-A/DS-B) sites of each river categories (i.e. dam-fragmented US and DS, dam-fragmented with SBT US, DS-B and DS-A, and free-flowing US and DS). Within-community (alpha) diversity was evaluated with Simpson’s diversity. Analysis of Variance (ANOVA) and Tukey post hoc test were used to test the significant difference between the Simpson’s diversity values of each river categories. The measure of dissimilarity between the species composition or the pairwise compositional dissimilarities (beta-diversity coefficient) between upstream and downstream sites within a river can be estimated using both qualitative and quantitative metrics. Qualitative metrics measure changes in communities based on presence or absence (incidence), while quantitative metrics measure differences in relative abundances between communities.

We calculated the Bray-Curtis dissimilarity index^60^ based on relative sequence abundance to estimate pairwise community distance. Ten jackknife replicates of the Bray-Curtis distance matrix were generated to provide a better estimate of the variability expected in beta diversity results by resampling the OTU table to 79,831 reads (site with the lowest read count). ANOVA and Tukey post hoc test were used to test the significant difference between the Bray-Curtis values estimated for each river categories (i.e. dam-fragmented US/DS, dam-fragmented with SBT US/DS-B and US/DS-A, and free-flowing US/DS). Variation between the macroinvertebrate composition among the rivers are inherent. However, community dissimilarity was calculated based on the dissimilarity between the up- and downstream communities with-in each river. The difference between the macroinvertebrates among the rivers does not affect the dissimilarity observed with-in the up- and downstream communities of each river. Additionally, a Pearson correlation analysis was performed to assess the relationship between the Bray-Curtis values and SBT operation/year. We hypothesized that Bray-Curtis dissimilarity decreases with increasing operation time, hence a one-tailed test was used. To assess the reliability of using sequence abundance to interpret alpha and beta diversities from the metabarcoding data, the relationship between sample abundance (morphologically-identified families/morpho-families) and sequence abundance (metabarcoding-identified taxa at the family level) were examined via linear regression analysis. Correlation was calculated using the F statistic for the regression equation log_10_ (Y + 1) = log_10_ (X + 1) + b, where Y is sequence abundance, X is sample abundance, and b is the intercept.

### Sediment Analysis

Top view (surface) photographs of the gravel bed were taken in triplicate in the field at each site to measure sediment size distribution using the software BASEGRAIN^61^. Characteristic grain size parameters, i.e. *D*_*m*_ (effective diameter) and *D*_*90*_ (particle size for which 90% of bed surface is finer), were used to represent each gravel bed. A paired sample t-test was used to compare the US and DS (DS-A/DS-B) sites of each river. All statistical analyses were performed in the XLSAT software package (Addinsoft XLSTAT ver. 19.01.40777).

### Data availability

Most data generated or analyzed during this study are included in the manuscript (and its Supplementary Information files). The raw and filtered sequence files, and the OTU dataset generated and analyzed, are available from the corresponding author on reasonable request.

## Electronic supplementary material

Supplementary Information
