# Supplementary material for: Ecological influence of sediment bypass tunnels on macroinvertebrates in dam-fragmented rivers by DNA metabarcoding
Source: Sci Rep. 2018 Jul 5;8:10185. doi: 10.1038/s41598-018-28624-2 (PMC6033945; doi:10.1038/s41598-018-28624-2)
Supplement: Supplementary file 1 — Supplementary Information [file 41598_2018_28624_MOESM1_ESM.pdf]

SUPPLEMENTARY INFORMATION

Ecological influence of sediment bypass tunnels on macroinvertebrates in dam-fragmented rivers by DNA metabarcoding

Joeselle M. Serrana[1, *], Sakiko Yaegashi[2], Shunsuke Kondoh[1], Bin Li[1], Christopher T. Robinson[3], and Kozo Watanabe[1]

[1]Department of Civil and Environmental Engineering, Ehime University, Bunkyo-cho 3, Matsuyama, Ehime, 790-8577, Japan

[2]Department of Civil and Environmental Engineering, University of Yamanashi, 4-3-11 Takeda, Kofu, Yamanashi 400-851, Japan

[3]Swiss Federal Institute of Aquatic Science and Technology (Eawag), Überlandstrasse 133, 8600 Dübendorf, Switzerland, and Institute of Integrative Biology, ETHZ, 8092 Zürich Switzerland

[*]corresponding author: joeselle.ms@gmail.com

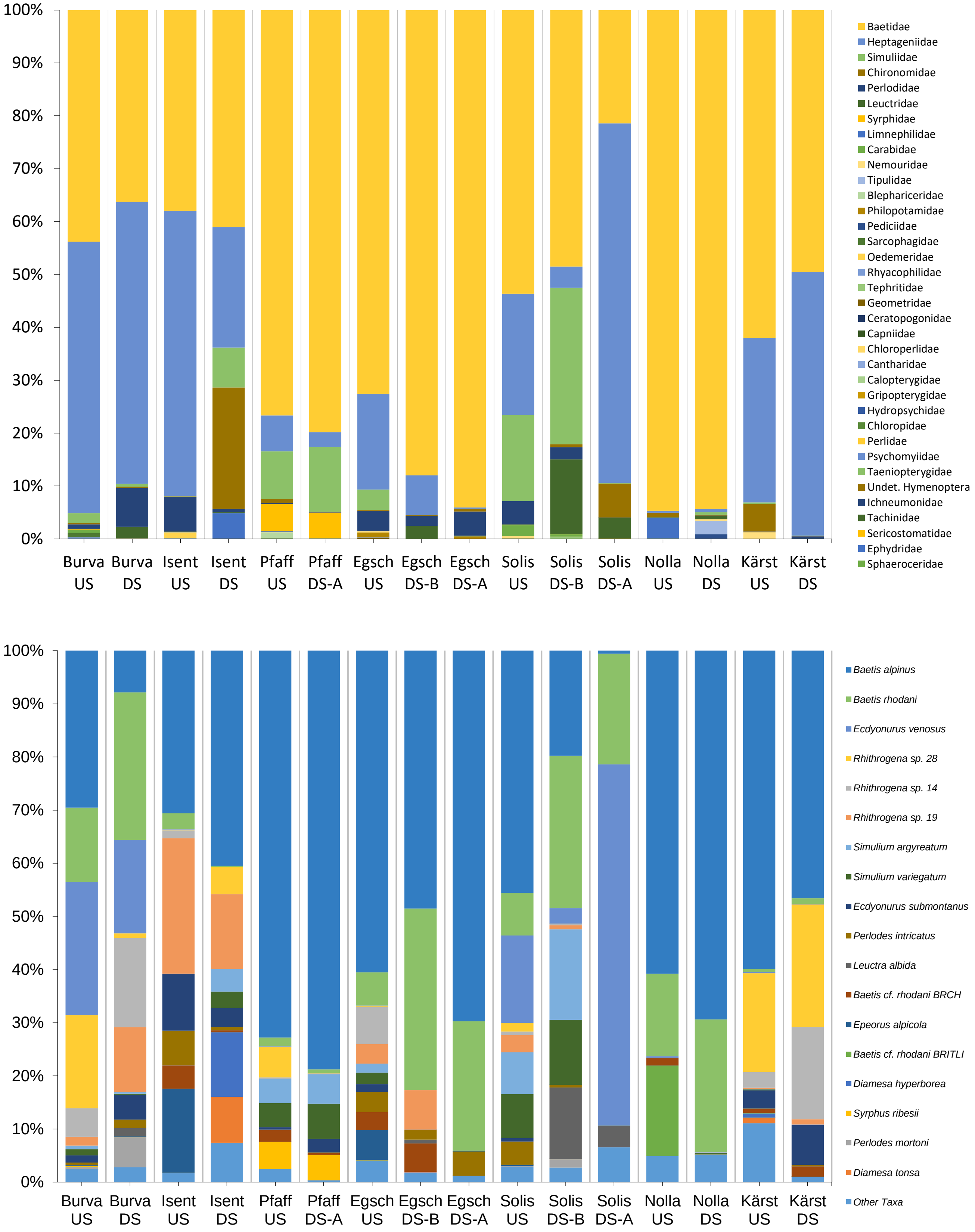

Figure S1. Relative abundance of metabarcoding-identified families and species (with ≥1% sequence abundance) of macroinvertebrates from the 16 sites. Site names are abbreviated; see map for full names of sampled dam sites.

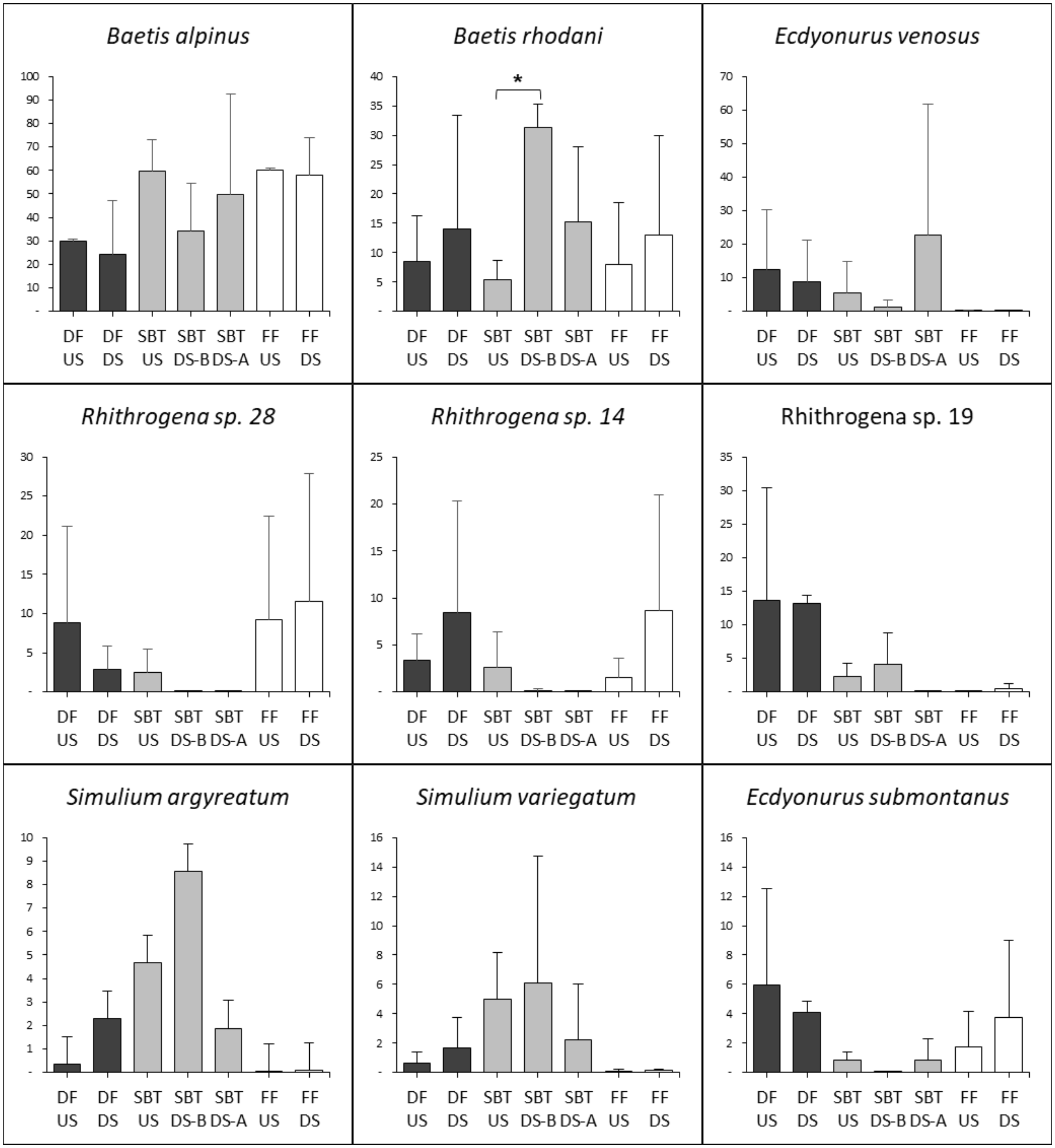

Figure S2. Relative abundance of species with >2% sequence abundance grouped per river categories. Axis values adjusted to show clear representation of percent abundance per group. US (upstream) and DS (downstream) (or DS-B/DS-A for SBT sites) with (*) indicating *p*-values < 0.05 after paired two-sample t-test. Site names are abbreviated; see map for full names of sampled dam sites.

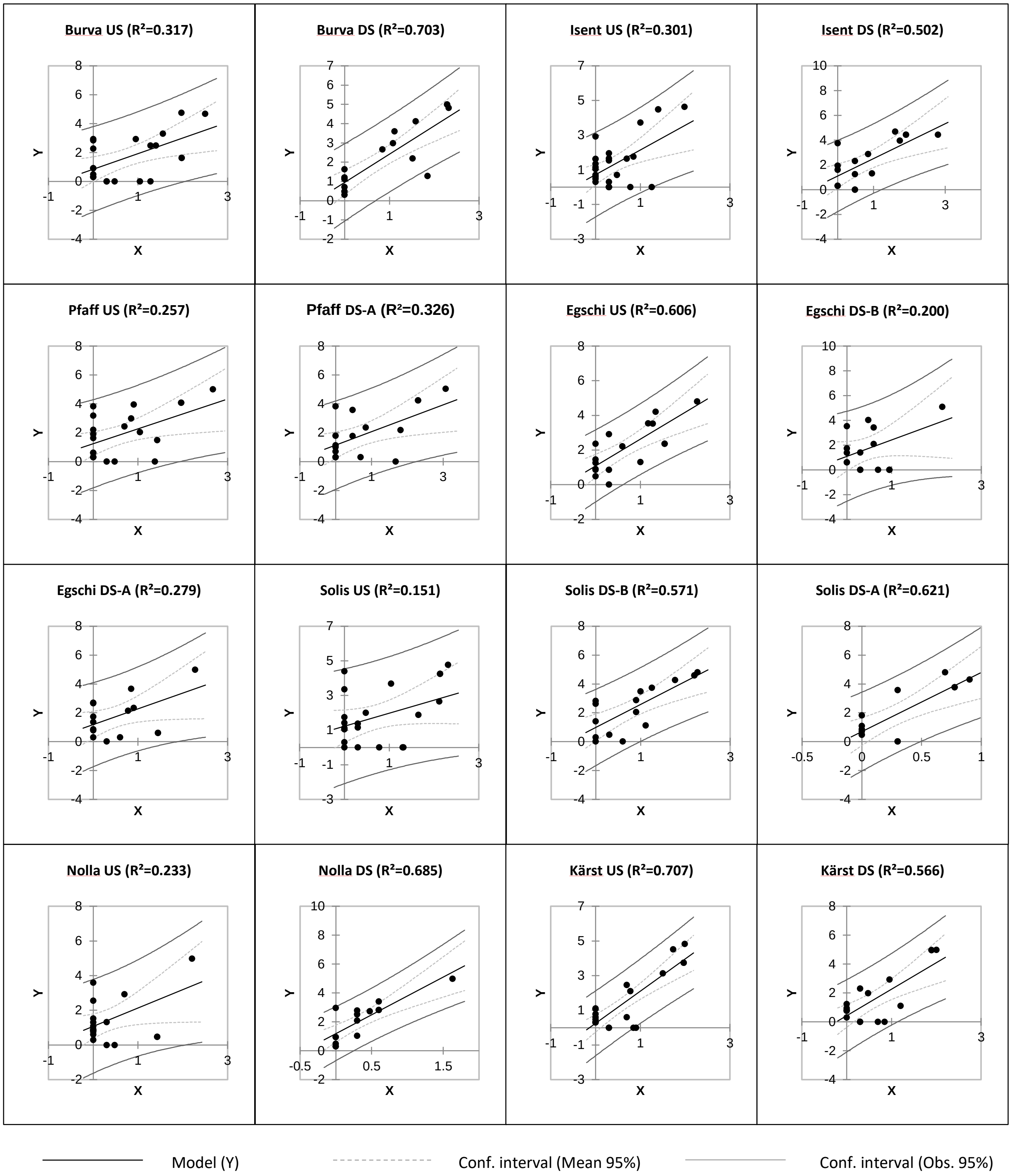

Figure S3. Correlations between the sample abundance (morphologically identified families or morpho-families) (X) and sequence/read abundance (metabarcoding-identified taxa at the family level) (Y) for each sample site, including false negative and false positive detection via linear regression analysis. Site names are abbreviated; see map for full names of sampled dam sites.

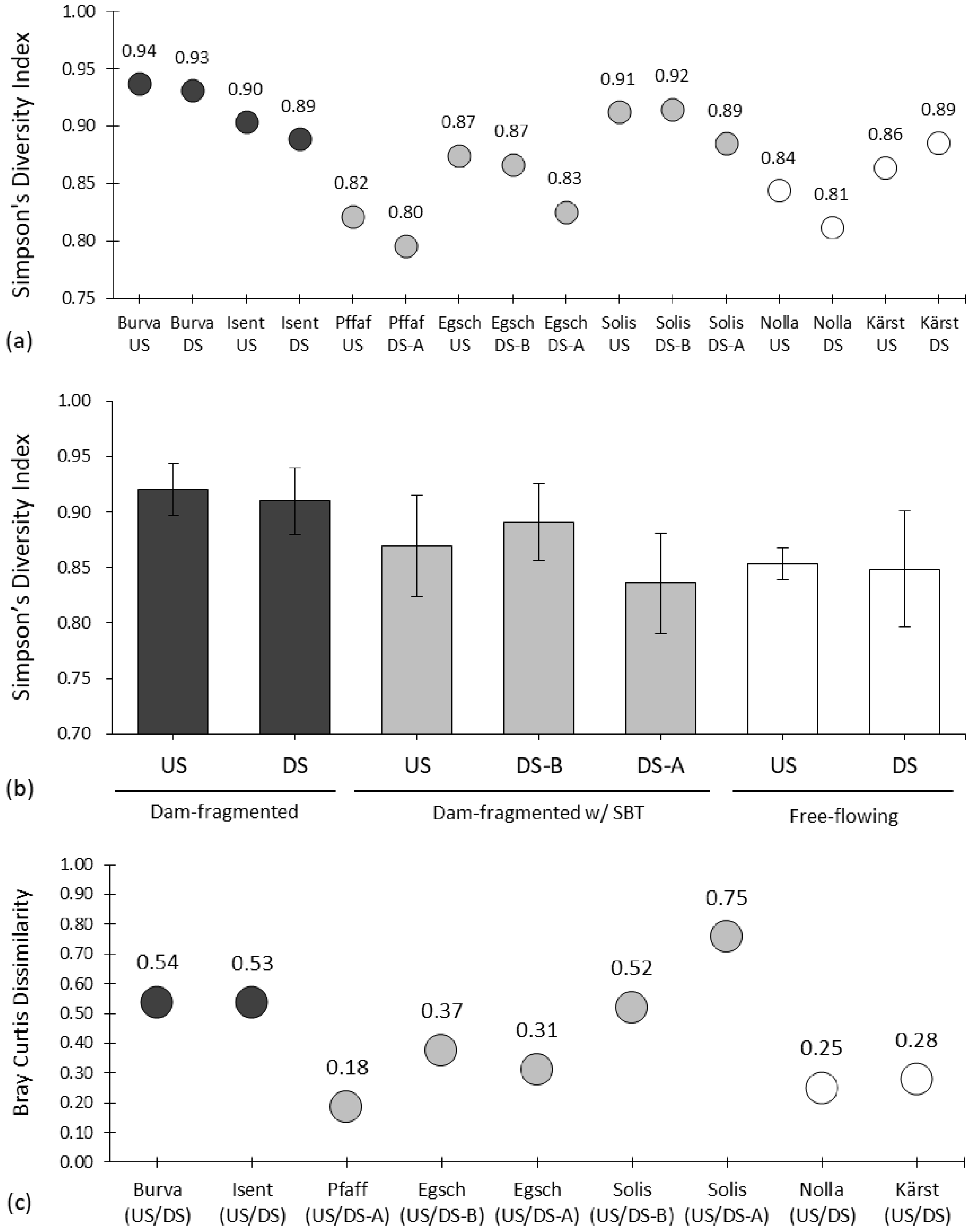

Figure S4. (a) Alpha diversity (Simpson's diversity index) for macroinvertebrate assemblages at the 16 sampling sites, and (b) alpha diversity of groups based on sampling site category. (c) Beta diversity (Bray-Curtis dissimilarity) of the macroinvertebrate communities between the up- (US) and downstream (DS) sites of dam-fragmented and free-flowing reaches, and between the up- (US) and downstream sites after the SBT outlet (DS-B/DS-A) at dam-fragmented sites with SBTs. Site names are abbreviated; see map for full names of sampled dam sites.

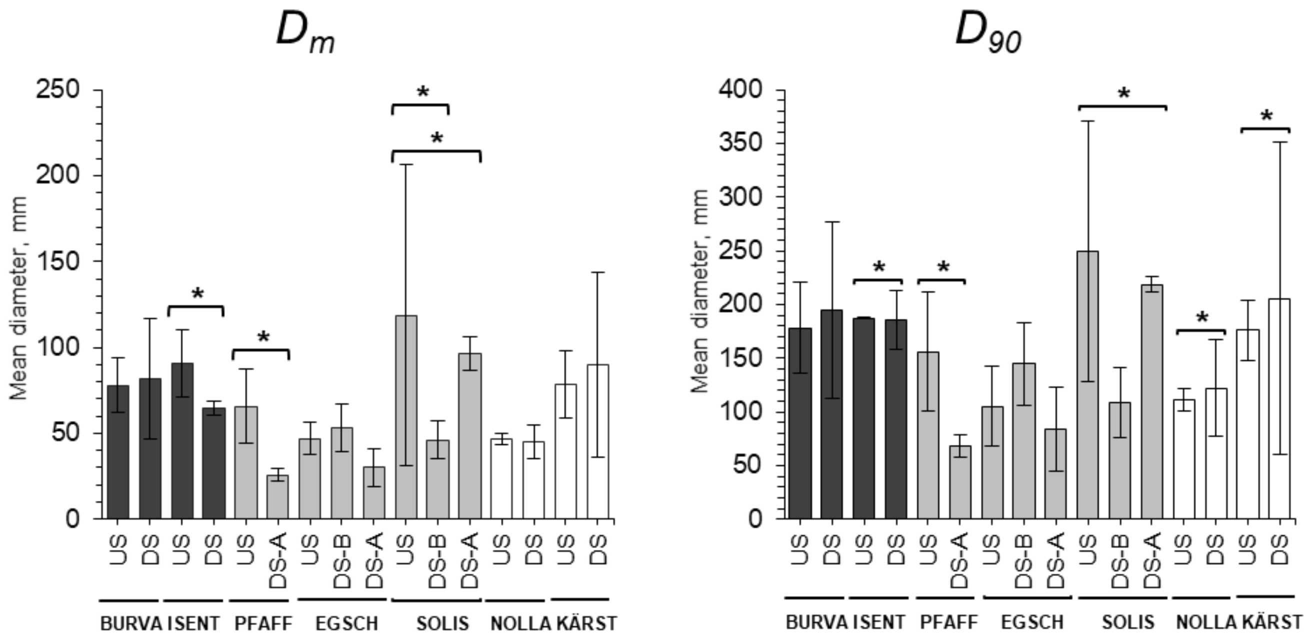

Figure S5. BASEGRAIN Analysis: Computed D_m and D_{90} values. US (upstream) and DS (downstream) (or DS-B/DS-A for SBT sites) with (*) indicating p-values < 0.05 after paired two-sample t-test. Site names are abbreviated; see map for full names of sampled dam sites.

Table S1. Percentage sample and/or read abundance of morpho-families detected/not detected by metabarcoding. SBT = sediment bypass tunnel.

Type	Sample Site	Sample Point	Morpho-family[†]	Sample Abundance (%)[‡]		Read Abundance (%)[§]		Linear Regression Analysis	
				Detected Morpho-family	False Negative	Detected Morpho-family	False Positive	R^2	Pr > F
Dam-fragmented	Burvagn Dam (*Gelgia River*)	US	14	94.85	5.15	98.39	1.61	0.32	0.01
		DS	9	100.00	0.00	99.95	0.05	0.70	< 0.0001
	Isenthal Dam (*Isenthalerbach*)	US	11	84.80	15.20	98.78	1.22	0.30	0.00
		DS	12	99.51	0.49	95.15	4.85	0.50	0.00
Dam-fragmented with SBT	Pfaffensprung Dam (*Reuss River*)	US	12	95.94	4.06	93.50	6.50	0.26	0.02
		DS-A	10	96.91	3.09	95.17	4.83	0.33	0.02
	Egschi Dam (*Rabiusa River*)	US	12	99.65	0.35	99.67	0.33	0.61	0.00
		DS-B	12	91.14	8.86	97.50	2.50	0.20	0.11
		DS-A	7	99.58	0.42	98.98	1.02	0.28	0.05
	Solis Dam (*Albula River*)	US	10	92.17	7.83	74.89	25.11	0.15	0.09
		DS-B	9	99.35	0.65	99.17	0.83	0.57	0.00
		DS-A	7	89.47	10.53	99.90	0.10	0.62	0.00
Free-flowing segment	*Nolla River*	US	7	98.45	1.55	95.56	4.44	0.23	0.05
		DS	7	100.00	0.00	99.10	0.90	0.68	0.00
	Kärstelenbach	US	12	95.02	4.98	99.96	0.04	0.71	< 0.0001
		DS	10	94.42	5.58	99.98	0.02	0.57	0.00

[†] Morphologically identified samples at the family level
[‡] Percentage abundance from the macroinvertebrate samples collected per point
[§] Percentage abundance of sequence reads from the metabarcoding analysis detected per point

Table S2. BASEGRAIN Analysis: Number of detected sediments per site and diameter (D) [mean (SD)]. SBT = sediment bypass tunnel.

Type	Sample Site	Sample Point	Detected Elements	D_{10} (mm)	D_{16} (mm)	D_{50} (mm)	D_{84} (mm)	D_{90} (mm)	D_m (mm)	D_{10}/D_{90}	σ_u
Dam-fragmented	Burvagn Dam (Gelgia River)	US	317	2.12 (0.41)	5.44 (1.05)	54.44 (11.22)	165.34 (40.43)	177.98 (42.46)	77.89 (16.03)	87.64 (32.66)	5.56 (1.08)
		DS	69	2.89 (0.92)	7.39 (2.34)	64.66 (20.32)	161.95 (98.39)	194.63 (82.16)	81.84 (35.06)	72.17 (32.42)	6.53 (3.48)
	Isenthal Dam (Isenthalerbach)	US	402	3.34 (1.69)	8.54 (4.33)	89.47 (52.90)	179.72 (0.84)	187.32 (0.52)	90.63 (19.66)	67.43 (35.04)	9.42 (9.00)
		DS	98	1.86 (0.43)	4.75 (1.09)	41.35 (12.82)	155.29 (12.06)	185.40 (27.25)	64.62 (3.73)	103.96 (29.69)	8.13 (3.23)
Dam-fragmented with SBT	Pfaffensprung Dam (Reuss River)	US	120	2.09 (0.39)	5.35 (0.99)	48.40 (15.64)	141.44 (52.37)	156.11 (55.32)	65.69 (21.70)	73.40 (12.47)	7.36 (3.76)
		DS-A	299	0.83 (0.14)	2.12 (0.36)	18.58 (1.88)	47.43 (7.54)	68.65 (10.56)	25.70 (3.49)	83.44 (8.70)	6.87 (3.68)
	Egschi Dam (Rabiusa River)	US	224	1.78 (0.02)	4.56 (0.05)	41.47 (3.02)	95.47 (32.80)	105.21 (37.47)	46.97 (9.50)	59.18 (21.71)	5.91 (2.07)
		DS-B	134	1.29 (0.28)	3.30 (0.71)	28.59 (6.06)	135.52 (40.57)	144.70 (38.31)	53.236 (13.77)	113.15 (19.73)	11.98 (10.12)
		DS-A	74	0.88 (0.25)	2.25 (0.64)	19.67 (3.57)	72.39 (37.00)	84.17 (39.07)	29.99 (11.08)	92.73 (27.34)	9.90 (8.28)
	Solis Dam (Albula River)	US	1372	3.82 (3.34)	9.81 (8.60)	135.00 (154.10)	221.07 (142.72)	249.25 (121.47)	118.76 (87.65)	80.69 (26.64)	8.71 (7.13)
		DS-B	92	1.74 (0.37)	4.44 (0.93)	40.92 (10.27)	86.14 (22.41)	108.39 (32.90)	46.12 (10.85)	62.26 (15.19)	5.78 (2.11)
		DS-A	179	2.77 (0.73)	7.10 (1.86)	80.79 (27.65)	202.67 (7.85)	218.75 (7.72)	96.40 (10.07)	83.37 (24.61)	7.51 (2.92)
Free-flowing segment	Nolla River	US	118	1.66 (0.10)	4.22 (0.27)	36.86 (1.54)	91.84 (9.11)	111.04 (10.51)	46.69 (3.21)	67.27 (7.710)	6.52 (3.06)
		DS	210	1.49 (0.17)	3.81 (0.42)	31.34 (5.65)	96.74 (24.03)	121.65 (45.02)	44.72 (9.74)	80.71 (22.30)	6.85 (2.69)
	Kärstelenbach	US	224	2.27 (0.73)	5.79 (1.85)	56.94 (21.55)	167.12 (24.28)	175.82 (28.38)	78.48 (19.39)	80.20 (12.46)	8.65 (5.60)
		DS	217	2.59 (0.84)	6.62 (2.14)	74.13 (24.92)	194.97 (139.24)	205.64 (145.84)	90.16 (53.79)	73.21 (29.15)	6.77 (2.20)

Table S3. Specifications of the three sediment bypass tunnels (SBTs) in Switzerland adopted from Sumi (2017)[1].

Dam	Completion Year	SBT Operation (yrs.)	Tunnel Specifications			Flow Discharge				Design Velocity (m/s)	Operation Frequency (per year)	Target Grain Size (mm)
			Cross Section Shape	Length (m)	Longitudinal Slope	Design Discharge (m3/s)	Specific Discharge (m3/s/km2)	Design Flood Return Period	Reservoir Maximum Outflow (%)			
Pfaffensprung	1922	92	Horseshoe shape 4.7mx4.8m	282	3.0% (1/33.3)	220	7.33	——	41.5	14	ca. 200 days	D_m: 250 D_{90}: 2,700
Egschi	1976	38	Circle 2r=2.8m	360	2.6% (1/38.5)	50	0.46	——	10.8	10	10 days	D_m: 100 D_{90}:300
Solis	2012	2	Hood-type 4.4mx4.7m	968	1.9% (1/52.6)	170	0.19	1/5 year	17.0	11	1-10 days	D_m: 60 D_{90}:150

Table S4. Bray-Curtis dissimilarity values and Sediment Bypass Tunnel operation data.

Dam	SBT Operation (in yrs.)	SBT Operation log-transformed	Bray-Curtis Dissimilarity (bet. US and DS/DS-A)	Reference
Burvagn	0	0.00	0.54	Assessed on the present study
Isenthal	0	0.00	0.53	
Pfaffensprung	92	1.97	0.18	
Egschi	38	1.59	0.31	
Solis	2	0.48	0.75	
Asahi	17	1.26	0.49	Kobayashi *et al.* (2016)[2]
Koshibu (Riffle)	0	0.00	0.58	
Koshibu (Pool)	0	0.00	0.73	
Pfaffensprung	93	1.97	0.56	
Solis	3	0.60	0.64	
Asahi	17	1.26	0.25	Auel *et al.* (2017)[3]
Koshibu	0	0.00	0.57	
Pfaffensprung	96	1.99	0.33	
Solis	2	0.48	0.31	

References

1. Sumi, T. Proceeding of the 2nd International Workshop on Sediment Bypass Tunnels, Kyoto University, Kyoto, Japan (ed. Sumi, T.) (2017).
2. Kobayashi, S., Auel, C., Sumi, T., & Takemon, Y. Recovery of Riverbed Features and Invertebrate Community in Degraded Channels by Sediment Supply through Bypass Tunnel. *Proceedings of the 12th International Conference on Hydroscience & Engineering for Environmental Resilience*, (2016).
3. Auel, C., Kobayashi, S., Takemon, Y., & Sumi, T. Effects of sediment bypass tunnels on grain size distribution and benthic habitats in regulated rivers. *International Journal of River Basin Management*, **15**(4), 1-46 (2017).
